# Orbital Langerhans Cell Histiocytosis: A Case Report

**DOI:** 10.7759/cureus.42773

**Published:** 2023-07-31

**Authors:** Tatsuro Yokoyama, Steffani Krista Someda, Hirohiko Kakizaki, Yasuhiro Takahashi

**Affiliations:** 1 Oculoplastic, Orbital and Lacrimal Surgery, Aichi Medical University Hospital, Aichi, JPN

**Keywords:** central nerve system involvement, chemotherapy, computed tomography, langerhans cell histiocytosis, orbit

## Abstract

An eight-year-old boy presented with a one-month history of left eyelid swelling. The patient was diagnosed with periorbital cellulitis at another clinic and was treated with oral antibiotics. However, the swelling did not subside. On initial consultation, the patient had left upper eyelid swelling with erythema. His extraocular muscle motility was normal, and the results of blood tests were unremarkable. A computed tomographic scan demonstrated a mass in the superior orbit with destructive changes in the left frontal bone. Histopathological examinations revealed a dense infiltrate of histiocytic cells. Immunohistochemical staining of the tumors for CD1a and Langerin was positive. A pathological diagnosis of Langerhans cell histiocytosis was made. Since orbital Langerhans cell histiocytosis has a high risk for central nervous system involvement, chemotherapy was the treatment of choice for any residual lesion to prevent sequelae to the central nervous system. At the six-month follow-up, the lesion did not recur, and the patient did not experience any central nervous system sequela.

## Introduction

Langerhans cell histiocytosis (LCH) is a rare inflammatory myeloid neoplasm that usually develops during childhood. LCH affects single or multiple organs and can be classified into three distinct types: single-system single site (SS-s), single-system multisite (SS-m), and multisystem (MS). Orbital involvement of LCH is rare, and a previous report showed only one (0.4%) of 250 biopsied orbital lesions in children was LCH [1]. Orbital LCH is recognized as one of the lesions with a high risk of central nervous system (CNS) involvement, causing systemic complications such as central diabetes insipidus, anterior pituitary hormone deficiency, and neurodegenerative disease [2]. Early diagnosis and therapeutic intervention are vital to prevent such CNS sequelae in cases with orbital involvement. However, orbital LCH is sometimes misdiagnosed as periorbital cellulitis because of the inflammatory response, which delays correct diagnosis and proper management.

Since the prognosis of SS-s LCH is generally good, excisional biopsy or curettage is chosen for the treatment of SS-s LCH [3]. However, chemotherapy is a recommended treatment option in patients with CNS-at-risk lesions to prevent CNS sequelae [3]. Herein, we report a pediatric patient with orbital LCH masquerading as periorbital cellulitis. Fortunately, the patient was immediately diagnosed as LCH upon consultation with our service, based on the clinical findings and computed tomography (CT).

## Case presentation

An eight-year-old boy presented with a one-month history of swelling in the left upper eyelid with no associated periocular pain or pain on eye movement. The patient was treated at an ophthalmology clinic with oral antibiotics for a few weeks, based on the diagnosis of left periorbital cellulitis. The swelling temporarily subsided but then relapsed shortly after. He had a prior history of ventricular septal defect and asthma. There was no remarkable family history. 

**Figure 1:**
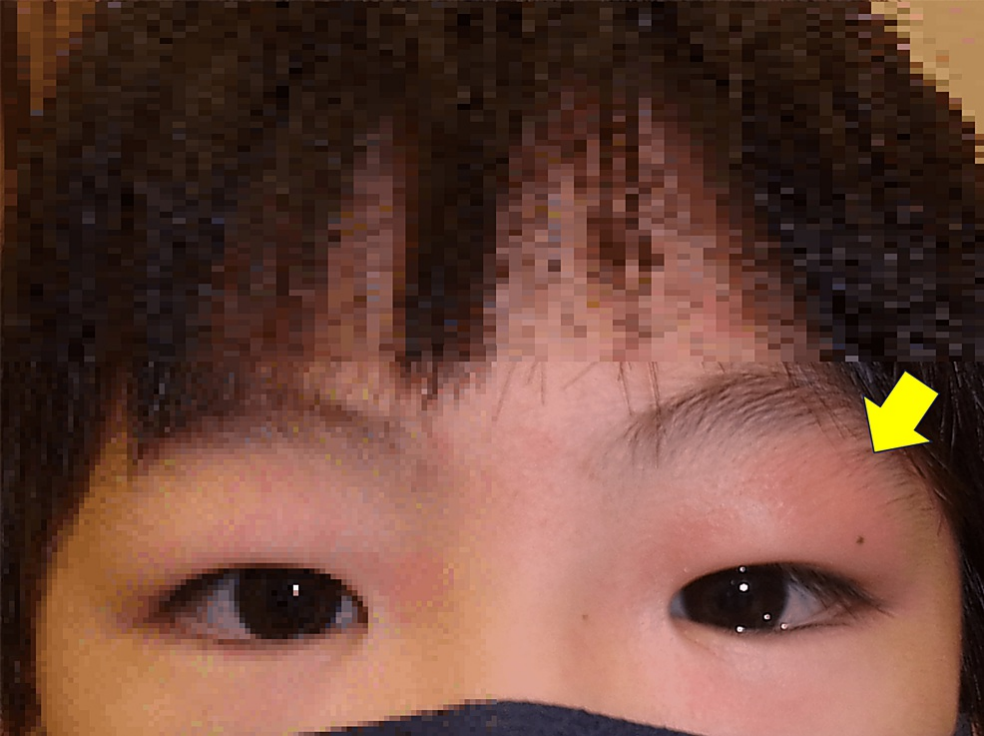
A face photo taken on initial examination The image shows swelling and erythema in the left upper eyelid (arrow).

On the initial consultation, the patient had noticeable swelling and erythema in the left upper eyelid without tenderness on palpation (Figure 1). A subcutaneous mass just below the left eyebrow was palpable. There was relative mechanical ptosis on the left upper eyelid, with a side-related difference in margin reflex distance of 0.5 mm. The visual acuity, intraocular pressure, pupillary reaction, ocular motility, and slit-lamp and fundus examinations were considered normal or unremarkable. The blood test results, including white blood cell count, C-reactive protein, soluble interleukin-2 receptor, and antinuclear antibodies, were within normal ranges. 

**Figure 2:**
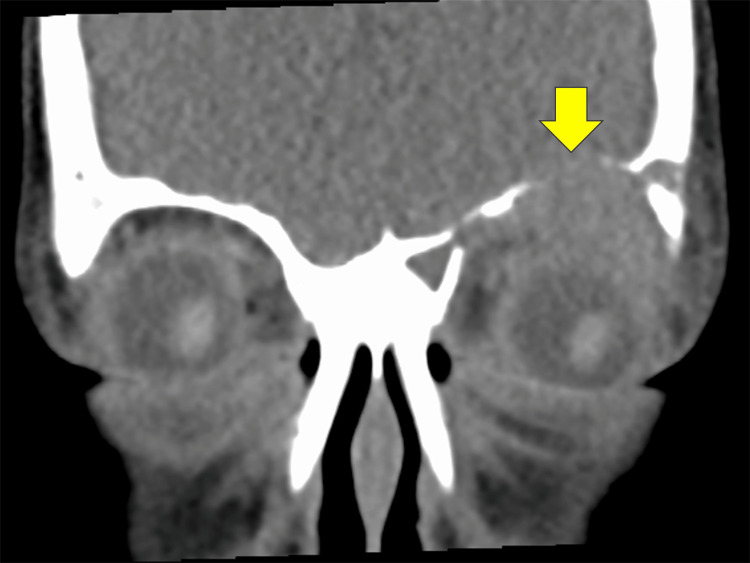
A coronal computed tomography image The image shows soft tissue mass in the superolateral orbit with an osteolytic lesion (arrow).

A CT scan demonstrated a mass in the superior orbit with destructive changes in the left frontal bone (Figure 2). There was an extension of the mass into the frontal sinus with the involvement of the lacrimal gland. Contrast-enhanced CT revealed minimal enhancement of the tumor. On magnetic resonance imaging (MRI), the tumor showed low intensity on T1-weighted images and high intensity on T2-weighted images. Based on the clinical findings and imaging studies, the patient was suspected to have orbital LCH instead of the previous diagnosis of orbital cellulitis. Hence, an incisional biopsy of the lesion was decided upon as the next step.

**Figure 3:**
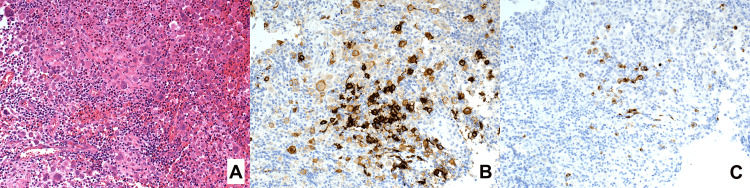
Findings of histopathological examinations Hematoxylin and eosin staining showing histiocytes with lymphocytes and eosinophils (A; magnification, ×200), and positive immunohistochemical staining for CD1a (B; magnification, ×200) and langerin (C; magnification, ×200).

A biopsy of the left orbital lesion was performed under general anesthesia by two of the authors (TY and YT) three days after the patient was referred to our department. The orbital lesion was removed as thoroughly as possible using an ultrasonic aspirator. Pathological examinations of the harvested specimen revealed a dense infiltration of histiocyte-like cells with infiltration of neutrophils, lymphocytes, and eosinophils (Figure 3A). Immunohistochemical staining of the tumor showed positive for langerin, CD1a, CD68, CD163, and S-100, but negative for ALK and BRAF (Figures 3B, 3C). The pathological diagnosis of LCH was made. The patient was then brought to a pediatrician for consult. On gadolinium-enhanced MRI, the size of the mass decreased, but a considerable size of the lesion was still left. Bone scintigraphy (99mTc-HMDP 212MBq) revealed accumulation only in the primary lesion.

Since orbital LCH has a high risk of CNS involvement, chemotherapy was the chosen treatment modality to address the remaining lesion and avoid CNS sequelae. Induction therapy, including cytosine arabinoside, vincristine, and prednisolone, was administered for six weeks, and maintenance therapy was given thereafter. At six-month follow-up, the lesion did not recur, and the patient did not experience any CNS sequela.

## Discussion

We report a pediatric case of orbital LCH who presented with left eyelid swelling and erythema, which led to a misdiagnosis of orbital cellulitis by the attending ophthalmologist. Although the incidence of LCH and orbital cellulitis are predominantly found in childhood [4], it is important to note that normal extraocular muscle motility and blood test results do not correspond to findings of orbital cellulitis. In addition, orbital LCH has a typical CT finding of a soft-tissue mass in the superolateral orbit centered in the frontal bones with osteolytic lesions [5,6], which was evident in our case. LCH produces interleukin-1, an osteoclast-activating factor, and prostaglandin E2, a potent inhibitor of bone formation [7]. The finding of bone destruction on imaging has certainly urged the authors to differentiate the tumor from other possible diseases, including malignancies, such as leiomyoma, myofibroma, solitary fibrous tumours, primary intraosseous haemangiomas, hemangiopericytoma, rhabdomyosarcoma, chondrosarcoma, myelogenous leukaemia, neuroblastoma, and osteosarcoma [8]. This subsequently led to the immediate biopsy of the lesion.

During the incisional biopsy of the left orbital lesion, we attempted to remove as much of the lesion as possible using an aspirator. However, a considerable size of residual tumor was left, which was confirmed on postoperative MRI. Since the patient had no other lesions throughout the rest of the body (SS-s LCH), re-curettage or re-aspiration of the lesion was a treatment option in our case. However, the risk of cerebrospinal fluid leakage outweighed the benefit of such a treatment option. Furthermore, chemotherapy was strongly recommended for cases with lesions at risk for CNS involvement. A previous study reported that more than 70% of patients with CNS-risking lesions received chemotherapy, and 144 of the 146 (99%) patients did not have active disease at the last follow-up [9]. Our case also obtained a good clinical course without any CNS sequela.

## Conclusions

We report a case of orbital LCH in a pediatric patient. Although eyelid swelling and erythema resembled orbital cellulitis, the typical CT finding of a soft tissue mass in the superolateral orbit with an osteolytic lesion led us to the early performance of the biopsy. Since orbital involvement is recognized as a CNS-risking lesion, chemotherapy was chosen to prevent CNS sequelae. A cooperative multidisciplinary approach involving not only general ophthalmologists and orbit specialists but also radiologists, pathologists, and pediatric oncologists is essential for the proper management of orbital LCH.
